# The annotation of repetitive elements in the genome of channel catfish (*Ictalurus punctatus*)

**DOI:** 10.1371/journal.pone.0197371

**Published:** 2018-05-15

**Authors:** Zihao Yuan, Tao Zhou, Lisui Bao, Shikai Liu, Huitong Shi, Yujia Yang, Dongya Gao, Rex Dunham, Geoff Waldbieser, Zhanjiang Liu

**Affiliations:** 1 School of Fisheries, Aquaculture and Aquatic Sciences, Auburn University, Auburn, Alabama, United States of America; 2 USDA-ARS Warmwater Aquaculture Research Unit, Stoneville, Mississippi, United States of America; 3 Department of Biology, Syracuse University, Syracuse, New York, United States of America; Ohio State University, UNITED STATES

## Abstract

Channel catfish (*Ictalurus punctatus*) is a highly adaptive species and has been used as a research model for comparative immunology, physiology, and toxicology among ectothermic vertebrates. It is also economically important for aquaculture. As such, its reference genome was generated and annotated with protein coding genes. However, the repetitive elements in the catfish genome are less well understood. In this study, over 417.8 Megabase (MB) of repetitive elements were identified and characterized in the channel catfish genome. Among them, the DNA/TcMar-Tc1 transposons are the most abundant type, making up ~20% of the total repetitive elements, followed by the microsatellites (14%). The prevalence of repetitive elements, especially the mobile elements, may have provided a driving force for the evolution of the catfish genome. A number of catfish-specific repetitive elements were identified including the previously reported *Xba* elements whose divergence rate was relatively low, slower than that in untranslated regions of genes but faster than the protein coding sequences, suggesting its evolutionary restrictions.

## Introduction

Eukaryotic genomes contain significant amount of repetitive DNA sequences, and the collective of the repeated sequences in an organism is known as the repeatome of the organism [1]. Such repetitive sequences were once thought to be junk DNA [2], but recent studies have indicated that they play important roles in propelling genome evolution and adaptation to environments [3–9]. The repeatomes of higher vertebrates, especially those of mammals, have been well studied, but their studies are limited for aquatic species.

Repetitive sequences can be generally divided into three major categories: the dispersed repeats such as transposable elements or transposons, tandem repeats, and high copy number genes [1]. Transposons are dispersed across genomes and their proportion are highly variable among genomes, ranging from 3% to 85% in terms of physical size [10–11]. For instance, the genome of *Utricularia gibba* contains only 3% of repetitive sequences [12–13], while the genome of maize contains over 85% transposable elements [14–15]. Based on their mechanisms of proliferation, transposons can be further classified into RNA-mediated Class I transposons and RNA-independent Class II DNA transposons. Class I transposons contain three main subclasses: short interspersed nuclear elements (SINEs), long interspersed nuclear elements (LINEs), and transposons with long terminal repeats (LTRs). The Class II transposons can be further divided into two classes based on their transposition mechanisms. The TIR-based Subclass-I elements such as piggyBacs, hATs, which are proliferated through the cut-and-paste mechanism; As well as non-TIR based Sub-class II DNA transposons such as Helitron, and Maverick; which are mobilize by rolling- circle replication via a single stranded DNA intermediate [16–17]. Transposons are capable of moving in the genome, and therefore, they are believed to be a major driving force for genome evolution [18–21].

Tandem repeats are individual repeats of DNA located adjacent to one another comprising variable numbers of nucleotides within each repeat sequence, and variable numbers of repeats such as microsatellites and satellites [22–24]. Tandem repeats are mostly presented in the centromeric, telomeric, and subtelomeric regions of chromosomes. In some cases, the tandem repeats can also make up large fractions of the genome [25–26]. The amplification and/or mutations of tandem repeats may also affect the genome by changing the genome structures or genome sizes [27–29], thereby affecting recombination of genomes, gene expressions, gene conversions, and chromosomal organizations [30–34].

High copy number genes such as ribosomal RNA (rRNA) genes or immunoglobulins also make up significant fractions of the repeatome. For instance, the copy numbers of rRNA genes can be as high as 4,000 copies, such as in the genome of pea (*Pisum sativum*) [35]. In *Saccharomyces cerevisiae*, a single cluster of rRNA can cover about 60% of the chromosome XII [36], thus it has been considered as the “king of the housekeeping genes” in terms of function and quantity [37]. Similarly, immunoglobulin genes have been found to be highly repetitive. For instance, the catfish IgH locus contains at least 200 variable (V) region genes, three diversity (D) and 11 joining (JH) genes for recombination [1, 38–39].

Channel catfish (*Ictalurus punctatus*) is a freshwater fish species distributed in lower Canada and the eastern and northern United States, as well as parts of northern Mexico. Its high tolerance and adaptability to harsh environments made it one of the most popular aquaculture species. In the United States, it is the primary aquaculture species, accounting for over 60% of all U.S. aquaculture production [40–41]. Its reference genome sequence with annotation of protein coding genes was published [42], but its repeatome was not fully characterized. Previous works reported the presence of an A/T-rich tandem Xba elements on the centromere regions but not in closely related species [43–44], and presence of dispersed SINE elements [45] and DNA transposons [46] in the channel catfish genome was also reported. Genomic sequencing surveys provided additional information about other repetitive sequences such as microsatellites, and transposons [47–52]. Here we annotated and characterized the repeatome of the channel catfish genome from sequences generated for whole genome sequencing.

## Material and methods

### Annotation of repetitive elements in channel catfish genome

The identification and annotation of the repetitive elements in the channel catfish genome assembly along with the degenerate sequences [42] were conducted using the RepeatModeler 1.0.8 package (http://www.repeatmasker.org/RepeatModeler.html) containing RECON [53] and RepeatScout [54]. The identified channel catfish repetitive sequences were searched against curated libraries and repetitive DNA sequence database Repbase [55] and Dfam [56] derived from RepeatMasker 4.0.6 package (http://www.repeatmasker.org/). To further determine characters of the repetitive elements classified as “Unknown” by the RepeatModeler, those sequences were first clustered by self-alignments via CD-HIT, with sequence identity cut-off set as 50% [57–58]. Then, the clustered sequences were searched against the entire NCBI Nucleotide collection database (nt) using BLASTN: 2.2.28+ with a relatively relaxed E-value (<10^−5^) to annotate the sequence with the best hit.

### The distribution and density of repetitive elements

The distribution frequency of the repetitive elements of DNA/TcMar-Tc1 as well as microsatellites and satellites sequences on the chromosomes were subtotaled and calculated by the location information and abundance information reported by the RepeatMasker. Their density on the chromosomes was presented as bp/MB. The heat map was plotted using the Heml1.0 [59].

### Divergence time of channel catfish and blue catfish

The divergence time and their 95% credibility intervals of channel catfish and blue catfish (*Ictalurus furcatus*) were calculated based on the divergence of cytochrome b genes with the calibration of fossil records. The substitution rate of cytochrome b was determined as normal distribution with mean of 1.05% and a standard deviation of 0.0105% [60]. In addition to channel catfish and blue catfish, we also used the cytochrome b sequences of blind cave fish (*Astyanax mexicanus*), common carp (*Cyprinus carpio*), and zebrafish (*Danio rerio*) for phylogenetic analysis (S1 File). The analysis was performed using the BEAST v.1.8.0 package [61]. Two independent runs were performed with 1,000 generations sampled from every 10 million generations for each dataset using MCMC chains [62]. The input files were constructed in BEAUTi, and the best substitution model was selected by Prottest 3.2.1 according to the alignments [63]. Model parameters consisted of a GTR+I+G model with a lognormal relaxed clock [64–65], the speciation birth-death process, and random starting tree were also applied in the phylogenetic analysis.

For the files of the resulting trees, we used the TreeAnnotator v1.8.0 to discard 10% of samples as burn-in and summarized the information of the remaining samples of trees onto a maximum clade credibility chronogram, and the results were viewed in Figtree with mean divergence times and 95% age credibility intervals.

To calibrate the divergence time of major clades for a better phylogenetic analysis, we have selected three teleost fossil records for calibration, the following node ages were set using lognormal priors:

1. Time of most recent common ancestor of *Ictalurus* (channel catfish and blue catfish), 19 MYR, (lognormal mean of 19 and standard deviation of 1.9), following Blanton and Hardman [66–67].
2. Time of most recent common ancestor of Characiformes (blind cave fish), 94 MYR, (lognormal mean of 94 and standard deviation of 9.4), with the fossil record discovered in Cenomanian [68–69].
3. Time of most recent common ancestor of Cypriniformes (common carp and zebrafish), 50 MYR, (lognormal mean of 50 and standard deviation of 5.0) with fossil discovered in Ypresian [70].

### Substitution rate of the Xba elements

The overall evolutionary dynamics can be referred from the average number of substitutions per site (K). The K was estimated from the divergence levels reported by Repeatmasker, using the one-parameter Jukes-Cantor Formula K = -300/4×Ln(1-D×4/300) as described in previous studies [71], where D represents the proportion of sites that differ between the fragmented repeats and the consensus sequence. For channel catfish and blue catfish Xba elements, the nucleotide substitution rate (r) was calculated using the formula r = K/(2T) [72], where T is the divergence time of channel catfish and blue catfish. To calculate the average K of the different types of repetitive elements, K of each element was multiplied by the length of the element, and the sum of all elements was divided by the sum of the total length of the elements.

## Results

### Annotation of repetitive elements in the channel catfish genome

**Fig 1:**
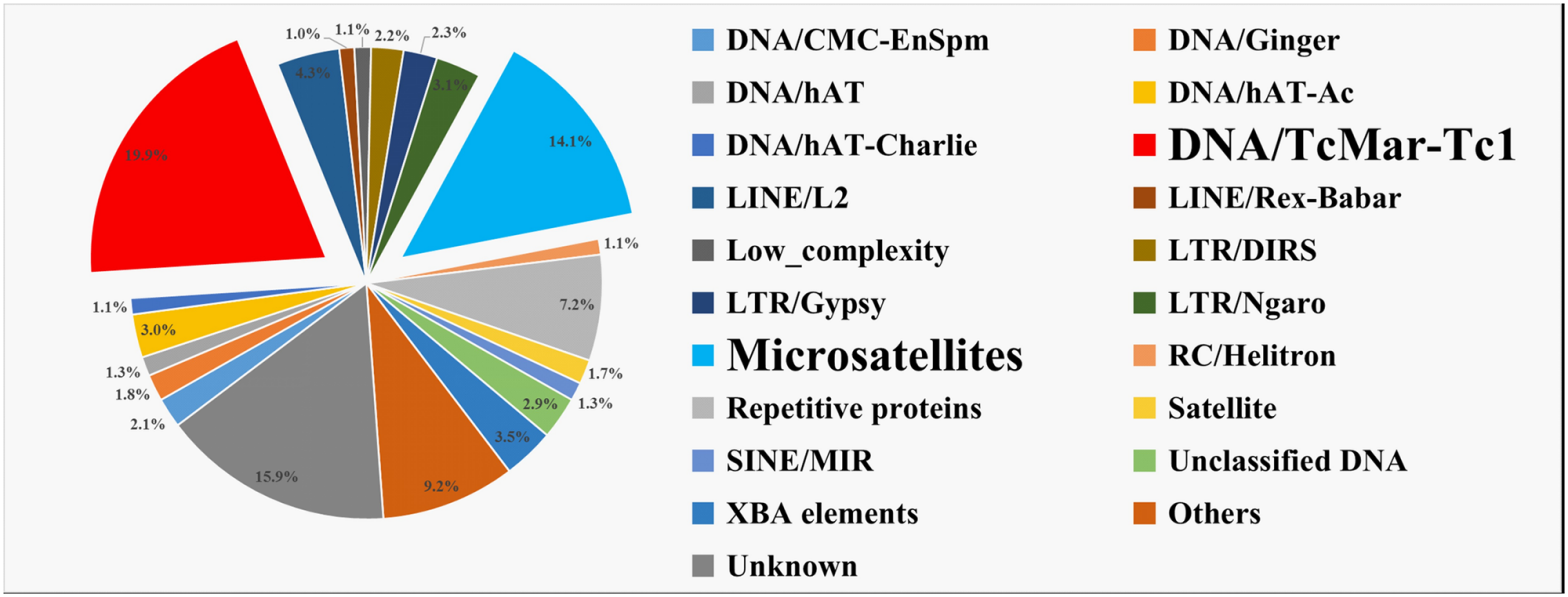
The proportion of major categories of repetitive elements within the channel catfish repeatome.

The major categories of the repetitive elements in the channel catfish genome are shown in Fig 1 and detailed in S1 Table and S2–S5 Files. The channel catfish genome harbored a total of 417.8 Mb of repetitive elements, accounting for 44% of the catfish genome. Of all the repetitive elements, 84.1% were annotated as known repetitive elements, while 15.9% were previously unclassified repetitive elements in the channel catfish genome. The known repetitive elements fell into 70 major categories, with the category of Tc1/mariner transposons accounting for the largest percentage (19.9% in repeatome, 8.8% in genome), followed by microsatellites (14.1% in repeatome, 6.2% in genome), repetitive proteins (7.2% in repeatome, 3.2% in genome), LINE/L2 (4.3% in repeatome, 1.9% in genome), Xba elements (3.5% in repeatome, 1.6% in genome), LTR/Nagro (3.1% in repeatome, 1.4% in genome), hAT/Ac (3.0% in repeatome, 1.3% in genome), unclassified DNA transposons (2.9% in repeatome, 1.3% in genome), LTR/Gypsy (2.3% in repeatome, 1.0% in genome), LTR/DIRS (2.2% in repeatome, 1.0% in genome), CMC-EnSpm (2.1% in repeatome, 0.9% in genome), Ginger (1.8% in repeatome, 0.8% in genome), satellite (1.7% in repeatome, 0.7% in genome), hAT (1.3% in repeatome, 0.6% in genome), SINE/MIR (1.3% in repeatome, 0.6% in genome), low complexity elements (1.1% in repeatome, 0.5% in genome), DNA/hAT-Charlie (1.1% in repeatome, 0.5% in genome), RC/Helitron (1.1% in repeatome, 0.5% in genome), and LINE/Rex-Babar (1.0% in repeatome, 0.5% in genome). All the remaining categories represented less than 1% each of the repetitive elements (S1 Table).

### The distribution of repeats cross genome

**Fig 2:**
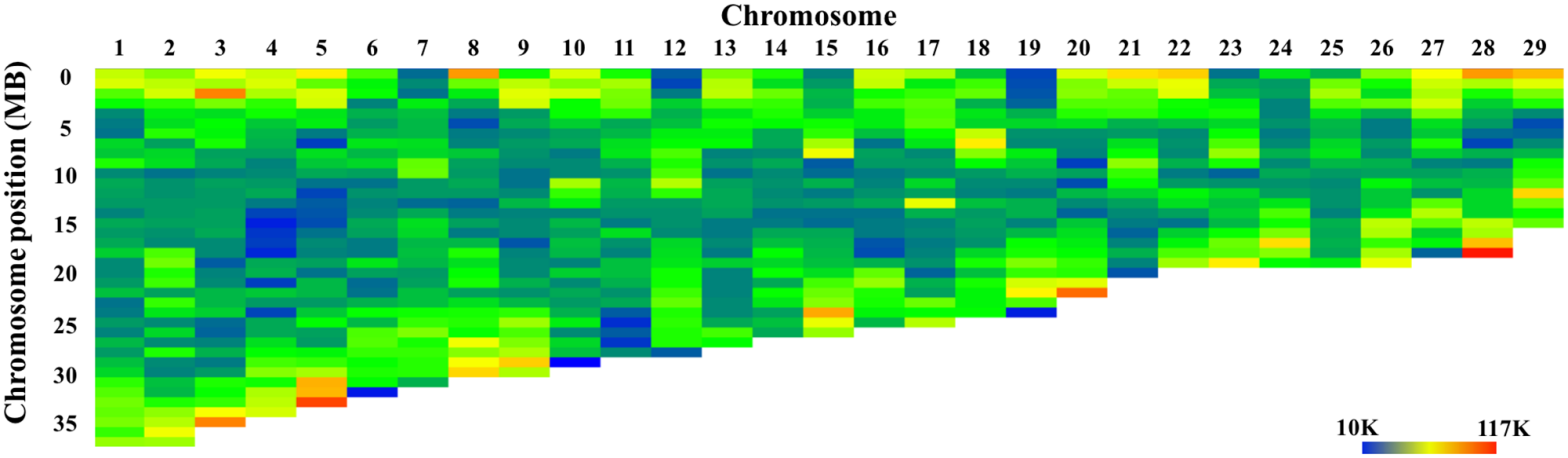
The distribution of Tc1/Mariner transposons cross channel catfish genome. Color key is indicated at the lower right of the figure, with blue color to indicate low and red color to indicate high levels of the transposons in the chromosomal regions. Each color bar represented a physical distance of 1 Mb DNA.

**Fig 3:**
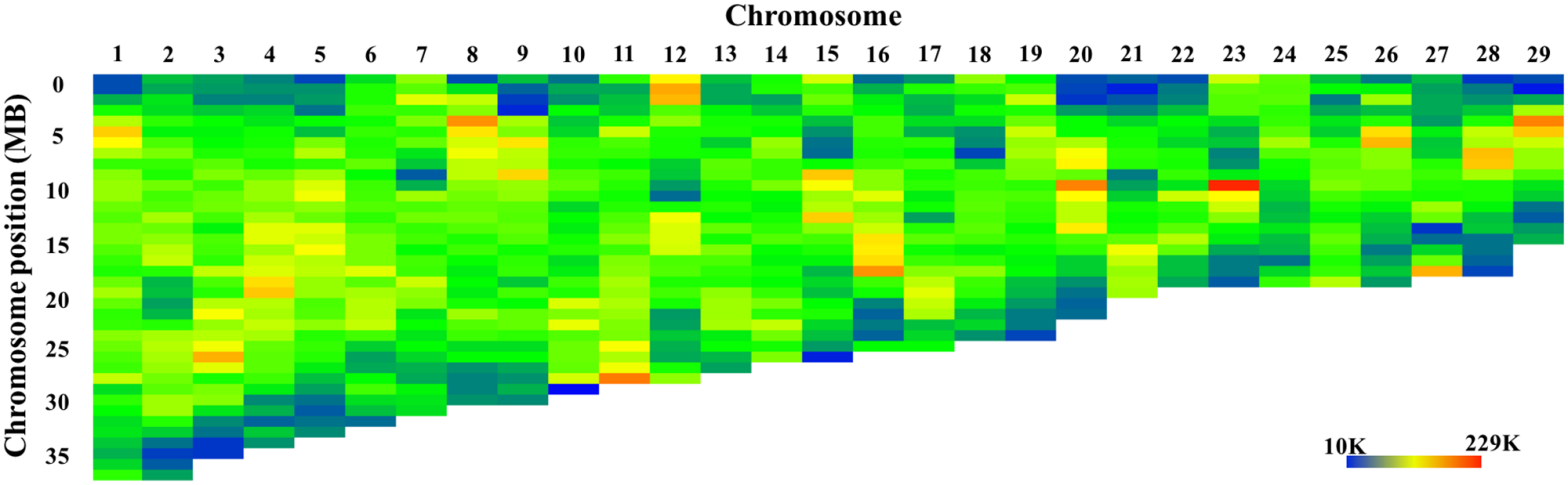
The distribution of microsatellites and satellites along the chromosomes of the channel catfish genome. Color key is indicated at the lower right of the figure, with blue color to indicate low and red color to indicate high levels of the microsatellites and satellites in the chromosomal regions. Each color bar represented a physical distance of 1 Mb DNA.

The Tc1/Mariner transposons are distributed cross the whole genome, with no major differences among chromosomes or among chromosomal regions within chromosomes (Fig 2). Among the annotated microsatellites, the dinucleotide microsatellites are the most abundant type, making up nearly 46% of the total annotated microsatellite sequences followed by tetra- and tri-nucleotide microsatellites, making up 18.6% and 13.6% of the total annotated microsatellites in length, respectively. As shown in Fig 3, the microsatellites and satellites are abundant on both ends of the chromosomes and some of them are distributed on the middle of the chromosomes. This is in consistent with previous results that telomere regions and centromere regions contain large part of short tandem repeats [73–75].

### Substitution rates

**Fig 4:**
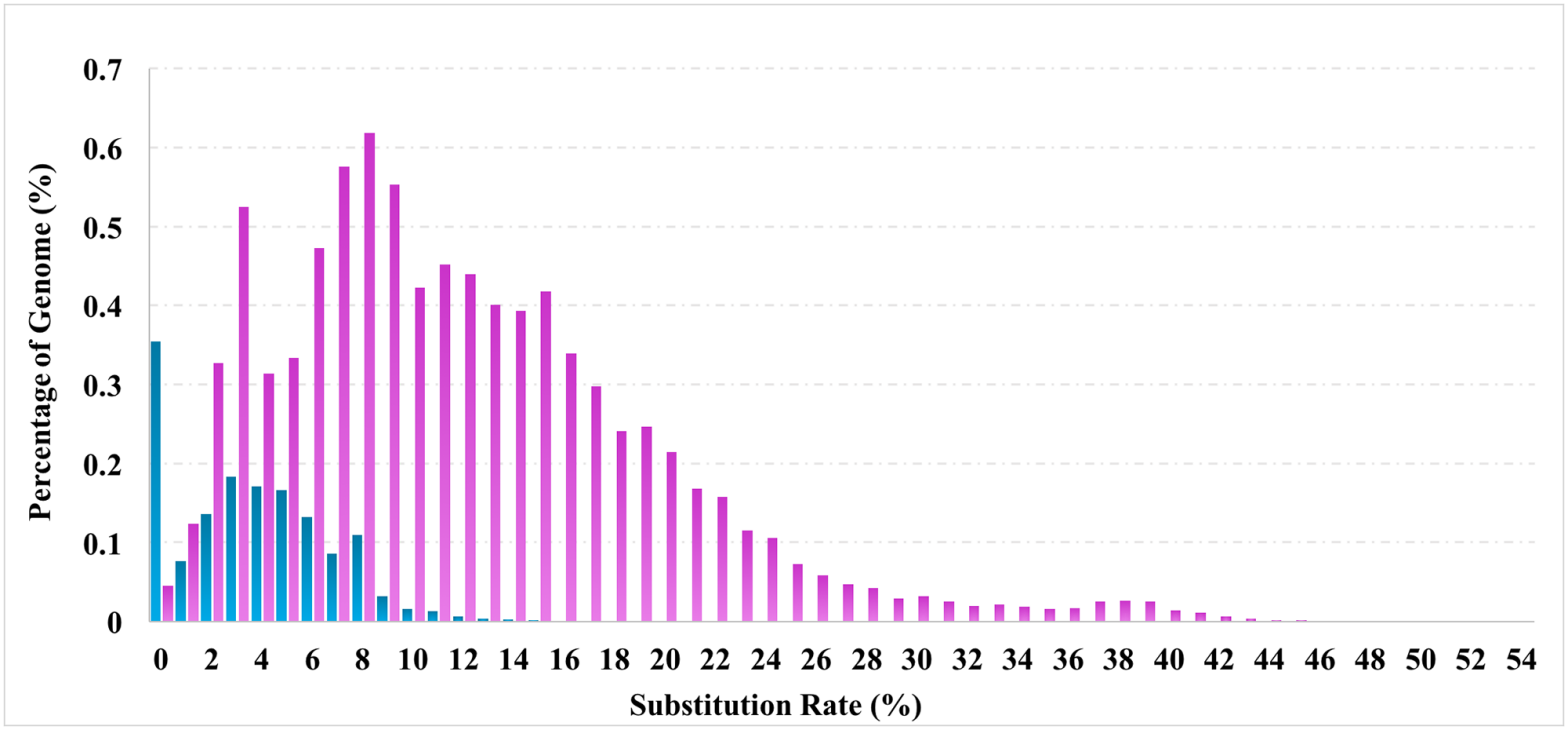
The divergence distribution of channel catfish Xba elements (blue) and DNA/TcMar-Tc1 transposons (pink). The X-axis represents the average number of substitutions per site (%), and the Y-axis represents the percentage sequences that comprise the whole genome (%).

The analysis of evolutionary rate of the unique Xba elements within catfish is useful to assess their limitations of evolution and assessment of their potential functions. The divergence analysis indicated that the Xba elements have a low mean Jukes-Cantor distance of 3.53, lower than the average Jukes-Cantor distance of 13.34 of the channel catfish reaptome. Meanwhile, compared with Xba elements, the substitution distribution of the catfish DNA/TcMar-Tc1 transposons, most prevalent in the catfish genome, are characterized not only by a broader distribution of divergence up to more than 50%, but also a larger mean divergence rate of approximately 12% (Fig 4). This indicated a long history of evolution as well as a more active evolutionary dynamics during the evolution of DNA/TcMar-Tc1 transposons in the catfish genomes, and recent acquisition of the Xba elements specific to the *Ictalurus* catfishes.

**Fig 5:**
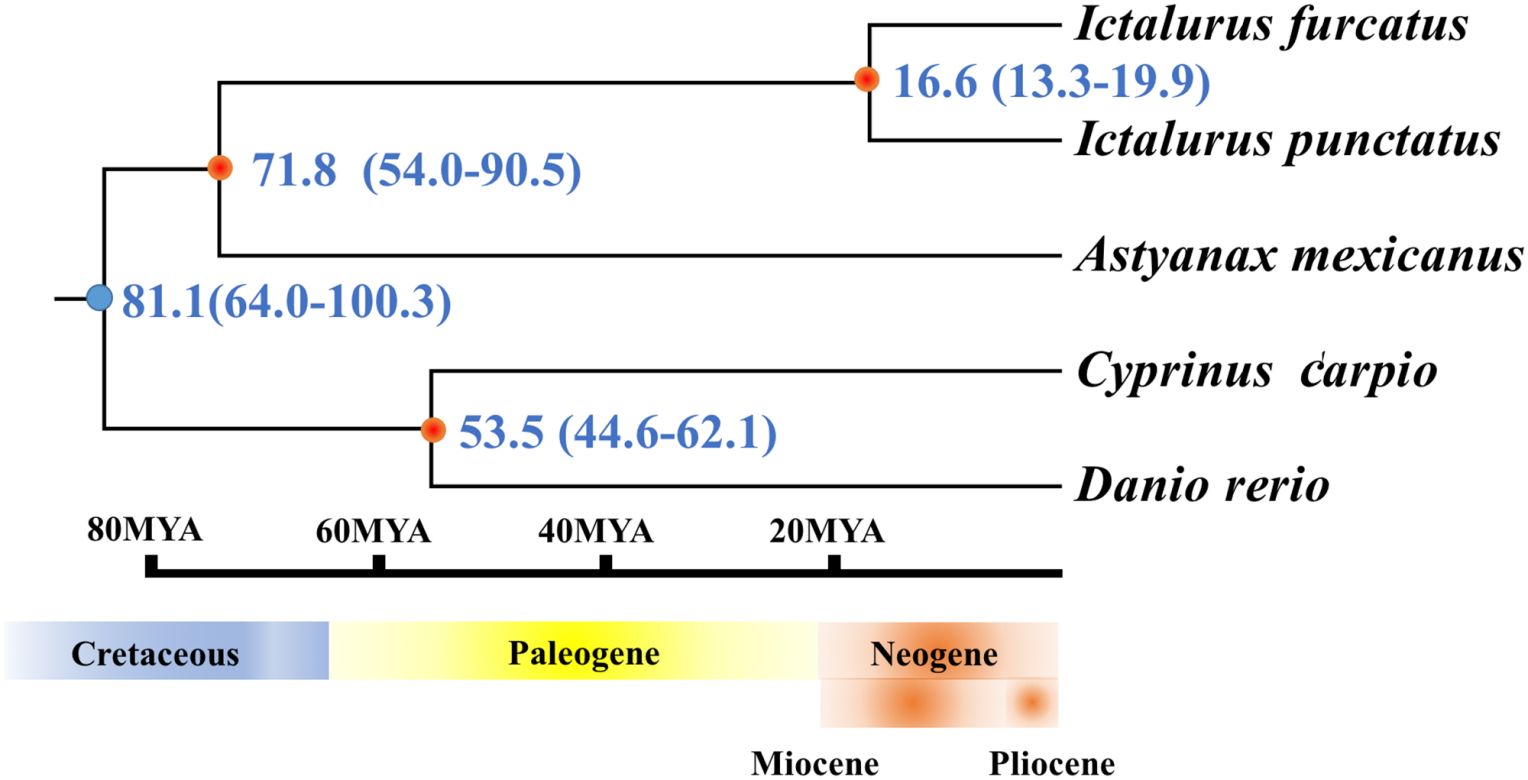
The divergence time of the channel catfish and blue catfish with fossil calibrations (orange nodes) based on the mitochondrial cytochrome b sequences.

The inference of divergence time of channel catfish and blue catfish are important for the calculation of the rate of nucleotide substitutions of their unique Xba elements. The maximum clade credibility chronogram analysis indicated that the channel catfish and blue catfish separated approximate 16.6 million years (Myr) ago, with a 95% age credibility intervals of 13.3–19.9 Myr (Fig 5). This is consistent with the earliest fossil record of the channel catfish discovered in Nebraska in the middle Miocene, and agreed with previous analysis of approximate 21 Myr of separation of channel catfish and blue catfish [66]. Based on the average number of substitution per site and the divergence time, the rate of nucleotide substitutions of the Xba elements was calculated as 8.9×10^−8^ to 1.3×10^−7^ substitutions per site per year. Meanwhile, based on the results of the previous research on differences of full length cDNA sequences between channel catfish and blue catfish [76], the rate of nucleotide substitution of Xba elements are higher than those in the open reading frame regions (2.5×10^−8^ to 7.6×10^−8^), but lower than those in untranslated regions (1.3×10^−7^ to 1.9×10^−7^).

### Novel repetitive elements in the catfish genome

Among the repetitive elements in channel catfish, there are still about ~16% of the repetitive sequences which cannot be annotated from neither the repetitive element databases nor the known non-redundant nucleotide database. Those sequences are rich in A/T (58%), the grouping of those sequences with more than 50% in similarity by CD-hit had grouped them into 215 categories (S2 Table). The top categories with over 500 Kb in length and their representative sequences on the genome are listed in Table 1. Those categories contain more than 15 Mb of the novel repetitive elements in length and most of them are also A/T enriched. Although there were no previous annotations of those repetitive elements, they may still have potential functions in the genome evolutions or biological processes regulations. Our work provides a brief classification of those repetitive elements (S2 Table). However, whether those sequences are generated internally or are “molecular parasites” from external environments, as well as the more detailed identifications and annotations of the functions of those novel repetitive elements still deserve further studies especially experiment demonstrations.

**Table 1:** The major novel repetitive elements and their characteristics in the channel catfish repeatome.

Size	AT content	Representative scaffold	Representative scf _start	Representative scf_end
1,417K	64.1%	IpCoco_scf00172	16,372,640	16,373,425
1,316K	45.2%	lcl|IpCoco_scf00610	3,078	6,525
1,081K	46.5%	IpCoco_scf00517	9,296,133	9,298,172
977K	46.0%	IpCoco_scf00474	141,399	143,674
963K	64.8%	IpCoco_scf00563_1077_652_655	1,427,889	1,431,117
924K	60.8%	IpCoco_scf00369	2,537,431	2,538,007
899K	50.1%	IpCoco_scf00203	3,637,116	3,638,889
881K	64.7%	IpCoco_scf00789	5,607	11,243
870K	64.9%	IpCoco_scf00540	509	4,548
842K	59.5%	IpCoco_scf00570_502_500	2,136,706	2,137,523
793K	44.5%	IpCoco_scf00419	7,439,807	7,440,803
690K	49.2%	IpCoco_scf00021	1,307,023	1,308,497
682K	47.2%	IpCoco_scf00077_78	260,739	262,006
630K	65.4%	IpCoco_scf00799	9,869	11,749
589K	53.3%	IpCoco_scf00354_353	643,250	644,319
546K	60.0%	IpCoco_scf00563_1077_652_655	1,372,133	1,373,248
522K	43.9%	IpCoco_scf00077_78	1,199,900	1,201,056
518K	66.6%	IpCoco_scf00172	2,251,598	2,252,801

## Discussion

### Repetitive elements in channel catfish

Using repetitive element databases combined with the nucleotide (nt) database, we identified, annotated, and characterized the repetitive elements in the channel catfish genome. Channel catfish harbors a large variety of repetitive elements in its genome, accounting for about 44% of its genome. The DNA transposons are the most abundant group of repetitive elements in the channel catfish genome, accounting for 15.9% of the catfish genome. These numbers are in line with our previous observations through genome sequence surveys [48, 52], but the data were analyzed from the whole genome and therefore is more complete.

The DNA/TcMar-Tc1 transposon sequences make up the highest percentage among Class II transposons in channel catfish genome, accounting for about ~20% of the total repetitive elements and are interspersed on the genome. The DNA-TcMAr/Tc1 is a typical ‘cut-paste’ transposon ([77]), which is prevalent in nature and can be transferred not only vertically but also horizontally cross species during evolution [78]. It is this character that allows DNA-TcMAr/Tc1 transposons to escape from the vertical extinction and being so abundant in nature [79–81]. Channel catfish is a freshwater benthopelagic species that inhabits in rapid fluctuating environments such as muddy ponds and rivers exposing to various biologic agents such as bacteria and viruses. Large amount of DNA/TcMar-Tc1 transposon footprints in channel catfish genome may indicate an external origin of parasitic transposable elements invasion to the genome during evolution [82]. As “parasitic” mobile elements, DNA transposons are known to be potent sources of mutation, and the long-time effective population shrinking in channel catfish can contribute to the evolution of more complex genomes such as more mobile elements or larger genome sizes [42, 83–84]. It is believed that the large amount of mobile transposons such as the DNA/TcMar-Tc1 can in turn contribute to the generation of novel genes and consequently facilitate considerably to species adaptations to novel environments [85–86]. Previous studies indicated that the transposition by a member of the Tc1/mariner family of transposable elements appears to have integrated in the duplicated Cμ region of the immunoglobulin [87]. Channel catfish is a quite hardy fish species that can survive in a wide range of environmental conditions [88]. It is also possible that the prevalent of DNA/TcMar-Tc1 sequences, as well as other transposons in channel catfish genomes, play important roles in their adaptations to environments. Currently, there are no specific hotspots of DNA/TcMar-Tc1 on each individual chromosome observed.

Considerable amount of tandem repeats, especially microsatellite sequences, were found in the channel catfish genome. As short tandem DNA repeats of 2–8 nt long are ubiquitous in nearly all eukaryotic genomes [89–91], the expansion of microsatellites is disputable but it is generally considered to be expanded through DNA polymerase slippage [92–94]. High content of microsatellites in catfish genomes compared with other freshwater teleost such as tilapia or medaka [95, 96], indicates a high level of DNA polymerase slippage, may suggest a relationship to the high magnesium concentration (meq/L) in the channel catfish tissue compared with other teleost [97–98]. It was speculated that the magnesium concentration can contribute to DNA polymerase slippage by stabilizing the hairpin structure [99]. However, DNA polymerase slippage is a very complicated process that can be affected by various conditions including the genome structures (such as GC content), DNA repair mechanisms, flanking DNA sequences (such as SINEs and LINEs), the centromere sequences and proteins involved in various DNA replication processes [100–109]. Whatever the mechanism is, high levels of microsatellites may help modulate the evolutionary mutation rate, thereby serving as a strategy to increase the species’ versatility under stressful conditions [110–111]. Our analysis of the distribution of the microsatellites and satellites indicates that those short tandem repeats were mostly presented on the telomere and the centromere regions of the chromosome, consistent with the previous analysis [112–116].

The catfish genome also contains a large fraction of repetitive proteins in the reaptome. The main types of repetitive proteins are related to the adaptive immunology and metabolism as previous analysis indicated [38]. This may indicate that the abundance of repetitive genes in the genome is an adaptation that meets the large demand of immune defenses. Remarkably, there are at least 3.8MB of protein coding repetitive domains that are identified to be related to immunoglobulins in the channel catfish genome (Table 2). This may suggest that the expansion of the immunoglobulin family in the channel catfish genome can be one of the mechanisms of its defense against various pathogens.

**Table 2:** The major repetitive protein domains characterized from the channel catfish repeatome.

Rank	Total Abundance	Gene Ontology
1	2,990K	Adaptive immunity, Immunity
2	1,046K	Adaptive immunity, Immunity
3	375K	Cellular morphogenesis.
4	353K	Nucleus, cell junction, nucleoplasm
5	255K	Integral component of membrane
6	232K	Unknown
7	214K	Kinase, Transferase
8	204K	DNA replication, mismatch repair
9	203K	Kinase, Transferase
10	174K	Transferase, Ubl conjugation, pathway, zinc binding
11	145K	GTP-binding, Nucleotide-binding
12	132K	Structural molecule activity
13	131K	Osteogenesis, Transcription regulation
14	129K	Cell adhesion
15	124K	Adaptive immunity, Immunity
16	118K	Adaptive immunity, Immunity
17	114K	Guanine-nucleotide releasing factor
18	109K	Integral component of membrane
19	109K	Hydrolase, Ligase, Oxidoreductase, Amino-acid biosynthesis, Histidine biosynthesis, Methionine biosynthesis, One-carbon metabolism, Purine biosynthesis, ATP-binding, NADP, Nucleotide-binding
20	108K	Hormone
21	108K	Zinc binding, Cytosol, Plasma membrane
22	107K	Nucleotide-binding, DNA integration
23	107K	Endopeptidase inhibitor, liver development
24	106K	ATP binding, protein serine/threonine kinase activity

### The divergence of Xba elements sequence in channel catfish

The Xba elements are a group of A/T-rich repetitive sequences that were found in channel catfish and blue catfish centromeres but not in closely related species such as white catfish (*Ameiurus catus*) and flathead catfish (*Pylodictus olivaris*) [43–44]. It is conserved among strains with minor changes in sequence identity and length, making it not only potentially important for genetic expression vectors but also of vital importance for the exploration of the channel catfish genome evolutions [43–44]. As the centromeres contain large amounts of DNA and are often packaged into heterochromatin, where the large-scale DNA sequences recombination and rearrangements varies greatly among phylogenetic related species [117–118]. The large amount of conservative Xba elements on centromere identified by fluorescencein situ hybridization [44] suggests a unique evolutionary status of the *Ictalurus* catfish. In addition, those centromeric repetitive sequences may be involved in centromere functions, such as kinetochore assembly and chromosome segregation during mitosis or meiosis [119–120], or even some epigenetic regulations [121].

Based on the number of substitutions per site and the divergence time, the rate of nucleotide substitutions of the *Xba* elements is calculated as 8.9×10^−8^ to 1.3×10^−7^ substitutions per site per year. Compared with the rate of nucleotide substitutions of full length cDNA calculated from the divergence level between the channel catfish and blue catfish [76], the rate of nucleotide substitutions of *Xba* elements is higher than that of the sequences in the open reading frames, but lower than those in untranslated regions. Slower rates of evolution suggest functional constraints [72]. The relatively slow evolutionary rate of *Xba* elements in catfish may indicate their potential functions, although unknown at present.

## Conclusion

In this study, we identified 417.8 MB of repetitive sequences in the channel catfish genome, among which 84% were annotated. Among the annotated repetitive element, the most prevalent was the DNA/TcMar-Tc1 transposons, making up ~20% of the repeatome, followed by microsatellite (14%). A number of catfish-specific repetitive elements were identified including the previously known Xba elements. This work represents the most comprehensive analysis of the repeatome of the channel catfish genome with the best available chromosomal assembly so far, and it should facilitate the annotation of various teleost genomes.

## Supporting information

S1 Table A list of the major categories of repetitive elements in channel catfish and their percentage in the total repeatome. (DOCX)

S2 Table A list of the clustering of novel repetitive elements in channel catfish, ranked by the number of contained sequences. (DOCX)

S1 File The cytb sequences along with the accessions for inferring the phylogenetic tree and divergence time. (FAS)

S2 File The GFF files of repeat annotations of channel catfish scaffold-1. (LZMA)

S3 File The GFF files of repeat annotations of channel catfish scaffold-2. (LZMA)

S4 File The GFF files of repeat annotations of channel catfish degenerate sequences-1. (LZMA)

S5 File The GFF files of repeat annotations of channel catfish degenerate sequences-2. (LZMA)
