# Supplementary material for: The annotation of repetitive elements in the genome of channel catfish (Ictalurus punctatus)
Source: PLoS One. 2018 May 15;13(5):e0197371. doi: 10.1371/journal.pone.0197371 (PMC5953449; doi:10.1371/journal.pone.0197371)
Supplement: S1 Table — (DOCX) [file pone.0197371.s001.docx]

**S1_Table. A list of the major categories of repetitive elements in channel catfish and their percentage in the total repeatome.**

| **Categories** | **Length** | **Percentage in repeatome** | **Percentage in genome** |
| --- | --- | --- | --- |
| DNA/CMC-EnSpm | 8,588,035 | 2.06% | 0.91% |
| DNA/Crypton | 242,175 | 0.06% | 0.03% |
| DNA/Crypton-V | 78,594 | 0.02% | 0.01% |
| DNA/Ginger | 7,637,015 | 1.83% | 0.81% |
| DNA/hAT | 5,487,708 | 1.31% | 0.58% |
| DNA/hAT-Ac | 12,360,979 | 2.96% | 1.31% |
| DNA/hAT-Blackjack | 897,878 | 0.21% | 0.10% |
| DNA/hAT-Charlie | 4,668,464 | 1.12% | 0.50% |
| DNA/hAT-hAT5 | 312,477 | 0.07% | 0.03% |
| DNA/hAT-hobo | 171,935 | 0.04% | 0.02% |
| DNA/hAT-Tip100 | 872,668 | 0.21% | 0.09% |
| DNA/hAT-Tol2 | 1,158,482 | 0.28% | 0.12% |
| DNA/IS3EU | 827,786 | 0.20% | 0.09% |
| DNA/Kolobok | 245,734 | 0.06% | 0.03% |
| DNA/Kolobok-T2 | 825,932 | 0.20% | 0.09% |
| DNA/Maverick | 2,710,651 | 0.65% | 0.29% |
| DNA/MULE-MuDR | 346,485 | 0.08% | 0.04% |
| DNA/MuLE-NOF | 71,642 | 0.02% | 0.01% |
| DNA/Novosib | 133,898 | 0.03% | 0.01% |
| DNA/P | 267,664 | 0.06% | 0.03% |
| DNA/PIF-Harbinger | 3,848,539 | 0.92% | 0.41% |
| DNA/PIF-ISL2EU | 358,050 | 0.09% | 0.04% |
| DNA/PiggyBac | 639,874 | 0.15% | 0.07% |
| DNA/Sola | 91,729 | 0.02% | 0.01% |
| DNA/TcMar | 535,998 | 0.13% | 0.06% |
| DNA/TcMar-ISRm11 | 85,918 | 0.02% | 0.01% |
| DNA/TcMar-Mariner | 90,702 | 0.02% | 0.01% |
| DNA/TcMar-Tc1 | 83,037,626 | 19.88% | 8.81% |
| DNA/TcMar-Tc2 | 149,381 | 0.04% | 0.02% |
| DNA/TcMar-Tigger | 950,714 | 0.23% | 0.10% |
| DNA/Zisupton | 238,823 | 0.06% | 0.03% |
| UnclassifiedDNA | 12,145,751 | 2.91% | 1.29% |
| LINE/I | 281,092 | 0.07% | 0.03% |
| LINE/I-Nimb | 355,326 | 0.09% | 0.04% |
| LINE/L1 | 1,937,508 | 0.46% | 0.21% |
| LINE/L1-Tx1 | 2,595,401 | 0.62% | 0.28% |
| LINE/L2 | 17,933,406 | 4.29% | 1.90% |
| LINE/Penelope | 422,511 | 0.10% | 0.04% |
| LINE/R1 | 1,362,877 | 0.33% | 0.14% |
| LINE/R2-Hero | 131,887 | 0.03% | 0.01% |
| LINE/Rex-Babar | 4,283,938 | 1.03% | 0.45% |
| LINE/RTE-BovB | 785,854 | 0.19% | 0.08% |
| LINE/RTE-X | 127,422 | 0.03% | 0.01% |
| UnclassifiedLINE | 111,578 | 0.03% | 0.01% |
| Low_complexity | 4,746,556 | 1.14% | 0.50% |
| LTR/Copia | 1,425,541 | 0.34% | 0.15% |
| LTR/DIRS | 9,233,420 | 2.21% | 0.98% |
| LTR/ERV | 224,039 | 0.05% | 0.02% |
| LTR/ERV1 | 3,025,892 | 0.72% | 0.32% |
| LTR/ERVK | 65,915 | 0.02% | 0.01% |
| LTR/Gypsy | 9,627,395 | 2.30% | 1.02% |
| LTR/Ngaro | 12,888,999 | 3.09% | 1.37% |
| LTR/Pao | 51,682 | 0.01% | 0.01% |
| LTR/Viper | 125,356 | 0.03% | 0.01% |
| UnclassifiedLTR | 508,483 | 0.12% | 0.05% |
| RC/Helitron | 4,491,267 | 1.07% | 0.48% |
| Retroposon | 230,172 | 0.06% | 0.02% |
| rRNA | 1,469,993 | 0.35% | 0.16% |
| Satellite | 6,945,285 | 1.66% | 0.74% |
| Microsatellites | 58,745,860 | 14.06% | 6.23% |
| SINE/5S | 1,193,623 | 0.29% | 0.13% |
| SINE/5S-Deu-L2 | 1,004,623 | 0.24% | 0.11% |
| SINE/Alu | 495,527 | 0.12% | 0.05% |
| SINE/MIR | 5,245,061 | 1.26% | 0.56% |
| SINE/tRNA | 125,260 | 0.03% | 0.01% |
| SINE/tRNA-L2 | 194,202 | 0.05% | 0.02% |
| SINE/tRNA-V | 1,944,266 | 0.47% | 0.21% |
| SINE/tRNA-V-CR1 | 431,196 | 0.10% | 0.05% |
| SINE/U | 133,552 | 0.03% | 0.01% |
| SINE? | 1,427,942 | 0.34% | 0.15% |
| snRNA | 81,984 | 0.02% | 0.01% |
| XBA | 14,700,253 | 3.52% | 1.56% |
| Repetitivegenes | 30,274,984 | 7.25% | 3.21% |
| Unknown | 66,328,071 | 15.88% | 7.04% |
