# Supplementary material for: The annotation of repetitive elements in the genome of channel catfish (Ictalurus punctatus)
Source: PLoS One. 2018 May 15;13(5):e0197371. doi: 10.1371/journal.pone.0197371 (PMC5953449; doi:10.1371/journal.pone.0197371)
Supplement: S2 Table — (DOCX) [file pone.0197371.s002.docx]

**S2_Table. A list of the clustering of novel repetitive elements in channel catfish, ranked by the number of contained sequences.**

| **Number of Sequences** | **Representative Sequence Scaffold** | **scf_start** | **scf_end** |
| --- | --- | --- | --- |
| 15,687 | IpCoco_scf00610 | 3,078 | 6,525 |
| 10,668 | IpCoco_scf00474 | 141,399 | 143,674 |
| 9,962 | IpCoco_scf00517 | 9,296,133 | 9,298,172 |
| 9,372 | IpCoco_scf00172 | 16,372,640 | 16,373,425 |
| 9,256 | IpCoco_scf00369 | 2,537,431 | 2,538,007 |
| 7,789 | IpCoco_scf00203 | 3,637,116 | 3,638,889 |
| 7,711 | IpCoco_scf00563_1077_652_655 | 1,427,889 | 1,431,117 |
| 7,314 | IpCoco_scf00540 | 509 | 4,548 |
| 6,816 | IpCoco_scf00419 | 7,439,807 | 7,440,803 |
| 6,593 | IpCoco_scf00021 | 1,307,023 | 1,308,497 |
| 6,040 | IpCoco_scf00789 | 5,607 | 11,243 |
| 5,982 | IpCoco_scf00570_502_500 | 2,136,706 | 2,137,523 |
| 5,665 | IpCoco_scf05654_567 | 71,525 | 74,021 |
| 5,432 | IpCoco_scf00563_1077_652_655 | 1,372,133 | 1,373,248 |
| 5,414 | IpCoco_scf00077_78 | 260,739 | 262,006 |
| 5,385 | IpCoco_scf00396 | 143,979 | 146,295 |
| 5,263 | IpCoco_scf00077_78 | 1,199,900 | 1,201,056 |
| 5,180 | IpCoco_scf00799 | 9,869 | 11,749 |
| 4,997 | IpCoco_scf00354_353 | 643,250 | 644,319 |
| 4,764 | IpCoco_scf00504 | 608,431 | 609,640 |
| 4,736 | IpCoco_scf00002_4_6 | 2,140,474 | 2,141,692 |
| 4,735 | IpCoco_scf00161_160 | 5,433,996 | 5,434,743 |
| 4,538 | IpCoco_scf00136_7095_137_139 | 9,991,070 | 9,991,859 |
| 4,535 | IpCoco_scf00141_142_968_5056_143 | 1,351,661 | 1,352,594 |
| 4,510 | IpCoco_scf00204 | 1,383,666 | 1,384,283 |
| 4,477 | IpCoco_scf00096 | 575,684 | 576,725 |
| 4,446 | IpCoco_scf00172 | 2,251,598 | 2,252,801 |
| 4,355 | IpCoco_scf00249 | 2,654,914 | 2,655,810 |
| 4,279 | IpCoco_scf00340_341_342 | 19,062,478 | 19,063,434 |
| 4,138 | IpCoco_scf00172 | 8,598,037 | 8,598,997 |
| 4,102 | IpCoco_scf05231 | 1 | 1,093 |
| 4,079 | IpCoco_scf00496_498_497 | 7,933,106 | 7,934,163 |
| 4,054 | IpCoco_scf02085 | 433 | 1,678 |
| 3,968 | IpCoco_scf00369 | 2,792,428 | 2,793,279 |
| 3,927 | IpCoco_scf00077_78 | 1,077,726 | 1,078,788 |
| 3,895 | IpCoco_scf00019_718 | 6,408,693 | 6,409,508 |
| 3,885 | IpCoco_scf00366 | 478,563 | 479,717 |
| 3,881 | IpCoco_scf00340_341_342 | 10,530,823 | 10,531,924 |
| 3,825 | IpCoco_scf00040 | 8,026,450 | 8,027,394 |
| 3,771 | IpCoco_scf00489_490_491 | 4,089,377 | 4,090,058 |
| 3,759 | IpCoco_scf00198_201 | 2,500,027 | 2,501,092 |
| 3,709 | IpCoco_scf00161_160 | 2,491,966 | 2,492,910 |
| 3,667 | IpCoco_scf00391_390 | 3,109,908 | 3,110,627 |
| 3,614 | IpCoco_scf00010 | 4,363,749 | 4,364,706 |
| 3,606 | IpCoco_scf00010 | 2,599,200 | 2,600,332 |
| 3,562 | IpCoco_scf00274_275 | 7,889,580 | 7,890,361 |
| 3,554 | IpCoco_scf00448 | 89,120 | 90,036 |
| 3,482 | IpCoco_scf00141_142_968_5056_143 | 11,727,384 | 11,728,311 |
| 3,474 | IpCoco_scf00096 | 309,930 | 310,766 |
| 3,472 | IpCoco_scf00136_7095_137_139 | 3,075,579 | 3,076,208 |
| 3,446 | IpCoco_scf00635_446 | 238,998 | 239,646 |
| 3,435 | IpCoco_scf00327_328 | 13,320,235 | 13,321,149 |
| 3,435 | IpCoco_scf00340_341_342 | 15,848,812 | 15,849,806 |
| 3,414 | IpCoco_scf00334 | 1,635,586 | 1,636,149 |
| 3,413 | IpCoco_scf00204 | 185,132 | 185,729 |
| 3,391 | IpCoco_scf00099_100 | 346,026 | 346,988 |
| 3,389 | IpCoco_scf00489_490_491 | 7,584,469 | 7,585,338 |
| 3,370 | IpCoco_scf00198_201 | 3,312,261 | 3,313,178 |
| 3,370 | IpCoco_scf00458_460 | 9,455,461 | 9,456,157 |
| 3,369 | IpCoco_scf00008 | 446,573 | 447,360 |
| 3,332 | IpCoco_scf00021 | 4,198,429 | 4,199,326 |
| 3,322 | IpCoco_scf00086 | 143,561 | 144,604 |
| 3,319 | IpCoco_scf00562_252 | 1,374,434 | 1,375,410 |
| 3,289 | IpCoco_scf00317 | 360,705 | 361,648 |
| 3,266 | IpCoco_scf00393_392 | 3,100,399 | 3,101,253 |
| 3,262 | IpCoco_scf00633 | 18,326 | 18,860 |
| 3,259 | IpCoco_scf00274_275 | 2,867,477 | 2,868,704 |
| 3,219 | IpCoco_scf00503 | 210,495 | 211,229 |
| 3,213 | IpCoco_scf00071 | 1,633,412 | 1,634,325 |
| 3,197 | IpCoco_scf00389 | 3,414,455 | 3,415,293 |
| 3,170 | IpCoco_scf00278 | 422,055 | 422,964 |
| 3,157 | IpCoco_scf04438 | 40 | 822 |
| 3,133 | IpCoco_scf00516 | 1,491,132 | 1,491,961 |
| 3,099 | IpCoco_scf00036 | 2,572 | 3,537 |
| 3,032 | IpCoco_scf00148 | 1,685,841 | 1,686,671 |
| 3,022 | IpCoco_scf00496_498_497 | 2,415,958 | 2,416,937 |
| 3,022 | IpCoco_scf00041 | 1,528,807 | 1,530,113 |
| 3,010 | IpCoco_scf00136_7095_137_139 | 7,830,924 | 7,831,811 |
| 3,008 | IpCoco_scf00525 | 3,198,193 | 3,199,630 |
| 2,951 | IpCoco_scf00525 | 6,200,193 | 6,200,986 |
| 2,950 | IpCoco_scf00327_328 | 2,324,626 | 2,325,657 |
| 2,919 | IpCoco_scf00042 | 1,116,339 | 1,117,093 |
| 2,917 | IpCoco_scf00286_7269 | 2,926,236 | 2,927,230 |
| 2,868 | IpCoco_scf00327_328 | 11,461,354 | 11,462,034 |
| 2,859 | IpCoco_scf00389 | 8,469,223 | 8,469,861 |
| 2,856 | IpCoco_scf00437 | 978,114 | 978,867 |
| 2,849 | IpCoco_scf04837 | 1 | 755 |
| 2,795 | IpCoco_scf00009 | 4,749,269 | 4,749,978 |
| 2,760 | IpCoco_scf00114_115 | 11,405,239 | 11,405,911 |
| 2,751 | IpCoco_scf00550_548 | 1,101,252 | 1,102,226 |
| 2,733 | IpCoco_scf00036 | 568,560 | 569,426 |
| 2,699 | IpCoco_scf00011 | 9,958,281 | 9,959,135 |
| 2,677 | IpCoco_scf00389 | 6,727,127 | 6,727,877 |
| 2,673 | IpCoco_scf00036 | 6,166 | 7,059 |
| 2,670 | IpCoco_scf00585 | 161,644 | 162,548 |
| 2,641 | IpCoco_scf00396 | 630,241 | 631,059 |
| 2,603 | IpCoco_scf00489_490_491 | 186,285 | 186,949 |
| 2,596 | IpCoco_scf00108_980 | 109,118 | 110,342 |
| 2,585 | IpCoco_scf00399 | 1,527,144 | 1,528,051 |
| 2,583 | IpCoco_scf05905 | 1 | 699 |
| 2,574 | IpCoco_scf00517 | 7,105,932 | 7,106,758 |
| 2,568 | IpCoco_scf00300_298_299 | 9,453,069 | 9,453,877 |
| 2,561 | IpCoco_scf00437 | 4,041,640 | 4,042,395 |
| 2,554 | IpCoco_scf00389 | 8,199,498 | 8,200,369 |
| 2,547 | IpCoco_scf00249 | 9,209,951 | 9,210,757 |
| 2,546 | IpCoco_scf00574 | 143,785 | 144,426 |
| 2,538 | IpCoco_scf00246 | 477,926 | 478,409 |
| 2,517 | IpCoco_scf00534 | 1,361,886 | 1,363,113 |
| 2,500 | IpCoco_scf00220 | 75,142 | 76,079 |
| 2,491 | IpCoco_scf00581_386 | 4,887,047 | 4,887,716 |
| 2,482 | IpCoco_scf00068_10963_69 | 2,015,920 | 2,016,548 |
| 2,468 | IpCoco_scf00232 | 8,205,457 | 8,206,098 |
| 2,462 | IpCoco_scf00936 | 9,673 | 10,464 |
| 2,410 | IpCoco_scf00092 | 2,673,153 | 2,674,146 |
| 2,406 | IpCoco_scf00021 | 5,350,196 | 5,351,010 |
| 2,387 | IpCoco_scf03832 | 621 | 1,104 |
| 2,344 | IpCoco_scf00550_548 | 1,159,155 | 1,159,846 |
| 2,293 | IpCoco_scf00496_498_497 | 10,977,345 | 10,977,986 |
| 2,280 | IpCoco_scf00136_7095_137_139 | 3,486,353 | 3,487,182 |
| 2,255 | IpCoco_scf00040 | 5,274,383 | 5,275,071 |
| 2,249 | IpCoco_scf00246 | 2,486,986 | 2,487,786 |
| 2,247 | IpCoco_scf00375 | 469,803 | 470,567 |
| 2,239 | IpCoco_scf00369 | 1,440,700 | 1,441,442 |
| 2,217 | IpCoco_scf00200 | 6,979,352 | 6,980,065 |
| 2,205 | IpCoco_scf00136_7095_137_139 | 5,679,024 | 5,680,155 |
| 2,187 | IpCoco_scf00428 | 7,162 | 7,938 |
| 2,179 | IpCoco_scf00609 | 147,467 | 148,139 |
| 2,137 | IpCoco_scf00389 | 3,057,922 | 3,058,552 |
| 2,034 | IpCoco_scf00391_390 | 3,173,448 | 3,173,715 |
| 2,017 | IpCoco_scf00366 | 1,994,548 | 1,995,380 |
| 2,010 | IpCoco_scf00200 | 11,537,699 | 11,538,622 |
| 2,006 | IpCoco_scf00369 | 9,305,743 | 9,306,545 |
| 1,998 | IpCoco_scf00315 | 1,172,781 | 1,173,466 |
| 1,989 | IpCoco_scf00171 | 1,308,407 | 1,309,107 |
| 1,970 | IpCoco_scf00141_142_968_5056_143 | 9,809,096 | 9,809,691 |
| 1,963 | IpCoco_scf00313_314 | 714,174 | 714,810 |
| 1,962 | IpCoco_scf00161_160 | 9,603,209 | 9,603,910 |
| 1,956 | IpCoco_scf00001 | 2,158,589 | 2,159,301 |
| 1,955 | IpCoco_scf00449 | 83,930 | 84,750 |
| 1,949 | IpCoco_scf00322 | 3,909,356 | 3,910,382 |
| 1,922 | IpCoco_scf04734 | 463 | 1,128 |
| 1,909 | IpCoco_scf00311_312 | 2,885,636 | 2,886,341 |
| 1,889 | IpCoco_scf00327_328 | 8,102,843 | 8,103,477 |
| 1,884 | IpCoco_scf00181_186 | 40,421 | 41,176 |
| 1,861 | IpCoco_scf00530_1311 | 162,525 | 163,199 |
| 1,852 | IpCoco_scf00437 | 6,867,161 | 6,867,864 |
| 1,814 | IpCoco_scf00327_328 | 13,947,769 | 13,948,538 |
| 1,811 | IpCoco_scf00550_548 | 2,870,570 | 2,871,331 |
| 1,791 | IpCoco_scf00340_341_342 | 15,931,004 | 15,931,648 |
| 1,790 | IpCoco_scf00525 | 11,756,306 | 11,756,809 |
| 1,787 | IpCoco_scf00171 | 1,219,854 | 1,220,499 |
| 1,780 | IpCoco_scf00771 | 25,166 | 25,765 |
| 1,762 | IpCoco_scf00200 | 14,190,348 | 14,191,003 |
| 1,751 | IpCoco_scf00096 | 814,481 | 814,993 |
| 1,720 | IpCoco_scf00136_7095_137_139 | 1,965,895 | 1,966,445 |
| 1,678 | IpCoco_scf00246 | 1,935,174 | 1,935,902 |
| 1,670 | IpCoco_scf00256 | 62,349 | 62,902 |
| 1,662 | IpCoco_scf00172 | 14,988,719 | 14,989,469 |
| 1,649 | IpCoco_scf00300_298_299 | 5,560,993 | 5,561,596 |
| 1,645 | IpCoco_scf00517 | 10,128,725 | 10,129,417 |
| 1,640 | IpCoco_scf00019_718 | 2,159,479 | 2,160,116 |
| 1,634 | IpCoco_scf00525 | 8,578,223 | 8,578,983 |
| 1,626 | IpCoco_scf00300_298_299 | 8,566,043 | 8,566,671 |
| 1,561 | IpCoco_scf00562_252 | 400,566 | 401,138 |
| 1,556 | IpCoco_scf00019_718 | 1,917,470 | 1,918,033 |
| 1,526 | IpCoco_scf00428 | 437,421 | 438,095 |
| 1,526 | IpCoco_scf00041 | 1,051,141 | 1,051,745 |
| 1,467 | IpCoco_scf00300_298_299 | 2,631,644 | 2,632,265 |
| 1,467 | IpCoco_scf00286_7269 | 2,067,689 | 2,068,092 |
| 1,454 | IpCoco_scf00352 | 1,929,317 | 1,929,879 |
| 1,452 | IpCoco_scf00274_275 | 12,438,671 | 12,439,359 |
| 1,451 | IpCoco_scf00419 | 5,751,012 | 5,751,689 |
| 1,440 | IpCoco_scf00420 | 12,159 | 12,798 |
| 1,405 | IpCoco_scf00581_386 | 211,372 | 212,113 |
| 1,389 | IpCoco_scf00496_498_497 | 6,569,791 | 6,570,391 |
| 1,387 | IpCoco_scf00114_115 | 11,583,522 | 11,584,112 |
| 1,384 | IpCoco_scf00455 | 3,789,094 | 3,789,670 |
| 1,372 | IpCoco_scf00011 | 28,281 | 28,982 |
| 1,368 | IpCoco_scf00393_392 | 2,768,677 | 2,769,258 |
| 1,364 | IpCoco_scf00151_2157_666_736 | 243,129 | 243,558 |
| 1,344 | IpCoco_scf00385 | 4,986,958 | 4,987,744 |
| 1,335 | IpCoco_scf00053 | 190,276 | 191,076 |
| 1,335 | IpCoco_scf00249 | 15,180,700 | 15,181,426 |
| 1,326 | IpCoco_scf00055 | 1,653,385 | 1,653,957 |
| 1,302 | IpCoco_scf00279 | 274,418 | 274,926 |
| 1,248 | IpCoco_scf00558 | 1,555,028 | 1,555,659 |
| 1,239 | IpCoco_scf00327_328 | 8,413,457 | 8,414,076 |
| 1,219 | IpCoco_scf00489_490_491 | 15,768,967 | 15,769,447 |
| 1,219 | IpCoco_scf00340_341_342 | 20,303,697 | 20,304,321 |
| 1,216 | IpCoco_scf04073 | 744 | 1,236 |
| 1,200 | IpCoco_scf00169 | 21,562 | 22,023 |
| 1,192 | IpCoco_scf00076 | 613,682 | 614,296 |
| 1,182 | IpCoco_scf00396 | 1,112,297 | 1,112,962 |
| 1,181 | IpCoco_scf00159 | 3,809,170 | 3,809,865 |
| 1,172 | IpCoco_scf00340_341_342 | 16,633,255 | 16,633,750 |
| 1,130 | IpCoco_scf00249 | 15,626,023 | 15,626,577 |
| 1,104 | IpCoco_scf00136_7095_137_139 | 695,027 | 697,319 |
| 1,097 | IpCoco_scf00385 | 3,752,059 | 3,752,742 |
| 1,067 | IpCoco_scf00169 | 44,619 | 45,147 |
| 1,053 | IpCoco_scf00517 | 4,189,110 | 4,189,611 |
| 1,031 | IpCoco_scf00114_115 | 8,376,101 | 8,376,678 |
| 997 | IpCoco_scf00008 | 315,955 | 316,492 |
| 981 | IpCoco_scf00826 | 15,287 | 15,773 |
| 918 | IpCoco_scf00203 | 2,217,944 | 2,218,386 |
| 914 | IpCoco_scf00369 | 7,486,442 | 7,486,954 |
| 885 | IpCoco_scf00279 | 699,557 | 700,099 |
| 875 | IpCoco_scf01014 | 1 | 479 |
| 848 | IpCoco_scf00114_115 | 8,292,985 | 8,293,540 |
| 812 | IpCoco_scf00011 | 7,410,043 | 7,410,515 |
| 779 | IpCoco_scf00102 | 20,267 | 20,625 |
| 767 | IpCoco_scf00233_234_235 | 293,553 | 293,962 |
| 747 | IpCoco_scf00389 | 1,861,045 | 1,861,731 |
| 684 | IpCoco_scf00204 | 317,790 | 318,339 |
| 645 | IpCoco_scf00370 | 3,751,623 | 3,752,048 |
| 574 | IpCoco_scf00711 | 8,014 | 8,428 |
| 136 | IpCoco_scf00009 | 3,058,070 | 3,058,197 |
